# Supplementary material for: Engineering intelligent chassis cells via recombinase-based MEMORY circuits
Source: Nat Commun. 2024 Mar 18;15:2418. doi: 10.1038/s41467-024-46755-1 (PMC10948884; doi:10.1038/s41467-024-46755-1)
Supplement: Supplementary file 1 — Supplementary Information [file 41467_2024_46755_MOESM1_ESM.pdf]

Engineering intelligent chassis cells *via* recombinase-based MEMORY circuits

Huang *et al*.

Supplementary Note 1: Defining an intelligent synthetic biological system

We define an intelligent biotic system as one or more chassis cells capable of (i) decision-making, (ii) coupled memory development, (iii) and communication between chassis cells and/or the host.

Supplementary Note 2: Inheritable synthetic memory

Decision-making is composed of one or more INPUT(s) mapped to an OUTPUT, such that the system can be reset upon the removal of the INPUT(s). In contrast, a synthetic memory operation is not reset upon the removal of cognate INPUT(s) – i.e., memory operations retain changes in the OUTPUT state upon the removal of the cognate INPUT(s). Canonical synthetic memory (type-I) is achieved by way of the regulation of a given recombinase, which is typically induced by a small molecule. Once matured (folded and assembled) the recombinase attaches to DNA elements *attB* and *attP* and results in reconfiguration of DNA. Recently, we reported a novel post-translational strategy for controlling recombinase function, termed interception (type-II), that expands the utility of recombinases for synthetic memory operations[1]. We define interception as the controlled blocking of any protein-DNA interaction (other than RNA polymerase) *via* a transcription factor (TF) that interacts with a cognate DNA operator pair in situ. Interception was achieved *via* strategically replacing a small segment of a recombinase attachment site with a DNA operator. We posit that mechanistically this results in the TF – when bound to operator DNA – sterically hindering a given recombinase from binding to a cognate attachment site. Correspondingly, under conditions in which the TF becomes unbound (i.e., induced) the said recombinase can attach to the DNA element and catalyze the reconfiguration of cognate DNA elements (e.g., resulting in deletion or inversion). Accordingly, this iteration of synthetic memory requires two parts: (i) an operation that regulates recombinase attachment post-translation, and (ii) a genetic circuit to define the memory function – i.e., the orientation and positioning of recombinase attachment sites *attB* and *attP*.

Supplementary Note 3: Mitigating crosstalk between inducible recombinase cassettes

Intriguingly, we observed two situations where transcription in one reading frame led to the activation of a gene located downstream of the active promoter, but in the opposite reading frame. We theorized that this antisense transcription from the first promoter could lead to the displacement of the TF bound to the second promoter, resulting in short windows for RNA polymerase (RNAP) to bind the second promoter and transcribe its downstream gene. Specifically, we saw that induction of the Int12 promoter resulted in slight activation of Bxb1, which was eliminated with the addition of a second terminator (L3S3P21) after the Int12 gene. A second, more prominent instance of this effect was seen with A118 expression (evidenced by the activation of the inversion GOF circuit) when promoters were located upstream of this inverted expression cassette. Unwanted A118 expression was mitigated with the addition of the L3S3P22 terminator upstream of the inverted expression cassette, corroborating our theory of antisense readthrough.

An additional challenge we encountered was the introduction of cryptic promoters when the six expression cassettes were combined into a single locus. Notably, we saw that placing the Int12 cassette after the A118 cassette, despite their promoters being in transcriptionally diverging directions and each gene having an upstream insulating terminator, led to activation of A118 in the absence of either inducer. A manual scan of the Int12 DNA sequence revealed a region with a 3 bp mismatch to the consensus σ^{70} promoter sequence (5'-TTGACA[N_{17}]TATAAT-3') oriented in frame with the A118 CDS (**Supplementary Fig. 2**). We posited that this led to unwanted transcription of A118, so we added a second terminator (L3S3P41) to block this cryptic promoter. This change successfully eliminated the expression of A118 in the absence of an inducer.

After insulation, the only instance of significant cross-induction was observed with A118 unexpectedly recombining ~9% of its targets in the presence of the Int12 inducer (3OC6 AHL). We confirmed that the Int12 inducer did not directly cause expression of A118 by interacting with PhlF and that Int12 was not responsible for recombining the A118 inversion GOF circuit (**Supplementary Fig. 3**). We posited that LuxR may exhibit bidirectional recruitment of RNAP given the high symmetry of its DNA operator, potentially leading to unwanted antisense transcription from its promoter. Interestingly, A118's regulator- PhlF- has previously been reported to provide a strong transcriptional roadblocking effect when used in genetic circuits[2]. These situations of unintended A118 expression suggest, however, evidence of PhlF being easily displaced from its promoter, potentially due to lower levels of PhlF expression used in this study.

Supplementary Note 4: Consideration of optimal recombinase expression levels

We believe it is worth emphasizing that the expression levels of recombinases were specifically tuned based on the inversion gain-of-function circuits. Our goal was to isolate expression variants that exhibited <5% recombination when uninduced and >95% recombination when induced. This is of note because each inversion GOF circuit features a strong constitutive promoter actively transcribing the 5' *att* site of the inversion circuit, which theoretically could impact the binding of a recombinase to

this *att* site and thus the efficiency of recombination. This may explain the slight variations in recombination performances when comparing inversion GOF vs. LOF or excision GOF vs. LOF circuits for a given recombinase. Simply put, varying the genetic context near *att* sites may result in subtle changes in recombinase efficiency and should be considered if the inversion and excision circuits are modified.

Supplementary Note 5: Design of PAM variants for CRISPRp

Given that the protospacer adjacent motif (PAM) sequence 5'-NGG-3' is required for effective sgRNA-dCas9 binding of DNA[3,4], we designed three different schemes for directing dCas9 to an *att* site: i) addition of a synthetic PAM sequence immediately upstream or downstream of the ~50-60 bp *att* sequence, ii) modification of the central core sequence of *attB* and *attP* sites to either CC or GG, creating two potential sgRNA binding sites, and iii) directing dCas9 *via* a naturally occurring PAM site located in an *att* sequence (**Supplementary Fig. 10**). The first design – i.e., adding synthetic PAM sequences adjacent to *att* sites – was most appealing as this would not modify the *att* sequence itself, and could provide up to four different targets per *attB*-*attP* pair (namely, one PAM at the 5' or 3' end of each *att* site). We designated these targets as P1-P4, moving from 5' to 3' in a given recombinase's inversion GOF circuit DNA sequence. Remarkably, we observed near-perfect protection from recombination for several of the tested sgRNA targets (**Fig. 6d**, also see **Supplementary Fig. 10**). While a specific design rule did not emerge regarding optimal placement, the design heuristic held given that all three schemes yielded protection levels of >95% when applied to certain recombinases. We did observe variable levels of protection from different sgRNA targets, likely due to individual sgRNA-dCas9 binding efficiencies and sgRNA misfolding, which has been reported previously[5-7]. Notably, the use of RNA folding tools did not reveal obvious connections between successful sgRNAs and proper folding. We also experienced instances of overprotection, specifically with the Int8 *att* core-targeting circuits. Initially, Int8 was unable to fully recombine its target when induced, which we posited was due to leaky sgRNA production. Weakening of the sgRNA promoter successfully restored Int8 recombination activity while still maintaining strong protection. In contrast, while we were able to demonstrate successful CRISPRp applied to A118, this required the use of an inducible dCas9, as we observed leaky A118 activity when using constitutive dCas9 (**Supplementary Fig. 11**). This collection of circuits highlights that CRISPRp is highly generalizable – i.e., in principle, can be applied to any recombinase system.

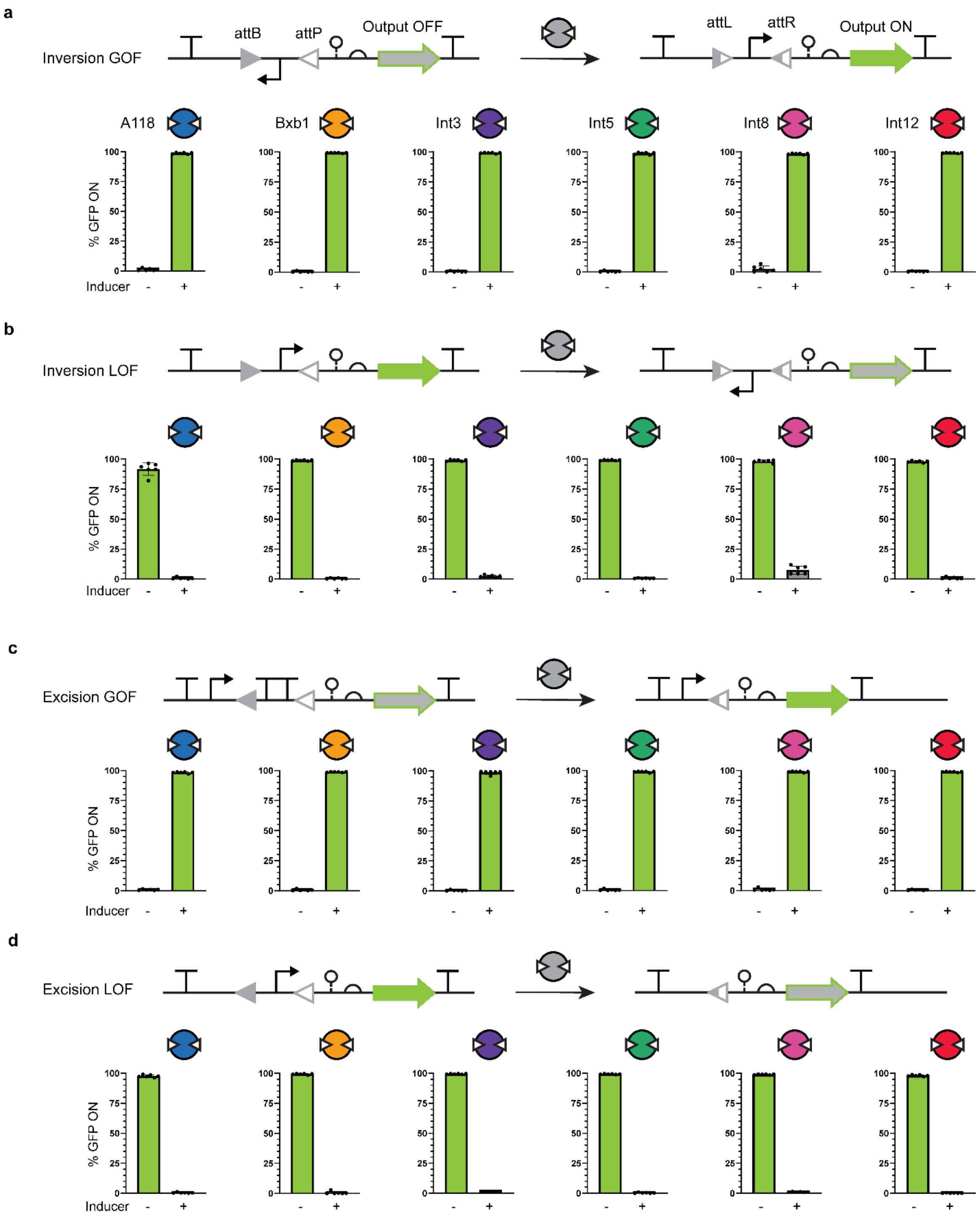

Supplementary Fig. 1 | Performance of BAC-based recombinase circuits in Marionette-Wild. a The performances of the inversion GOF circuits are shown when the individual recombinases are harbored on the BAC. **b** The performances of the inversion LOF circuits are shown when the individual recombinases are harbored on the BAC. **c** The performances of the excision GOF circuits are shown when the individual recombinases are harbored on the BAC. **d** The performances of the excision LOF circuits are shown when the individual recombinases are harbored on the BAC. All data represent experiments performed using Marionette-Wild. Source data are provided as a Source Data file. Data represent the average of $n = 6$ biological replicates, with groups of three taken on two separate days. Error bars correspond to the SEM of these measurements.

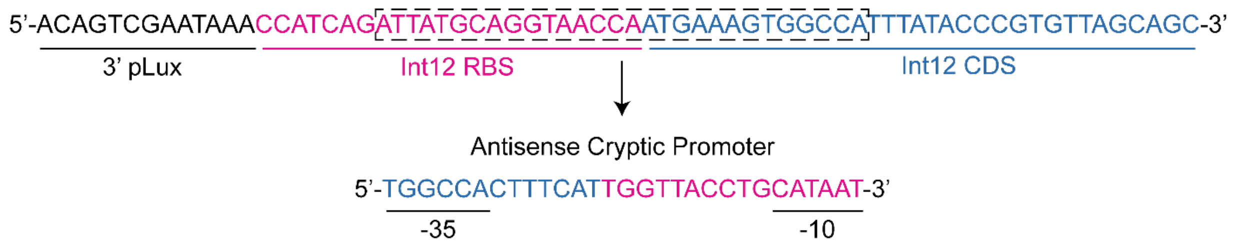

Supplementary Fig. 2 | A cryptic promoter identified in the Int12 sequence. Key sequence features of the Int12 cryptic promoter are shown. The boxed sequence is an antisense promoter containing only a 3 bp mismatch with the consensus sigma 70 promoter sequence.

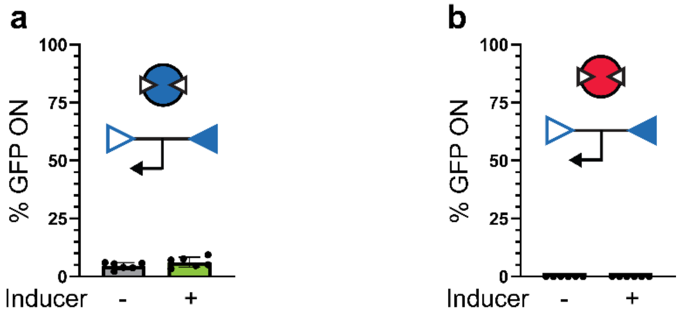

Supplementary Fig. 3 | Orthogonality between A118 and Int12. a PhlF is not induced by 3OC6 AHL. Marionette-Wild was transformed with the A118-harboring BAC and A118 inversion GOF plasmid and grown in the presence and absence of 3OC6 AHL. **b** Int12 does not recombine A118 *att* sites. Marionette-Wild was transformed with the Int12-harboring BAC and A118 inversion GOF plasmid and grown in the presence and absence of 3OC6 AHL. Source data are provided as a Source Data file. Data represent the average of $n = 6$ biological replicates, with groups of three taken on two separate days. Error bars correspond to the SEM of these measurements.

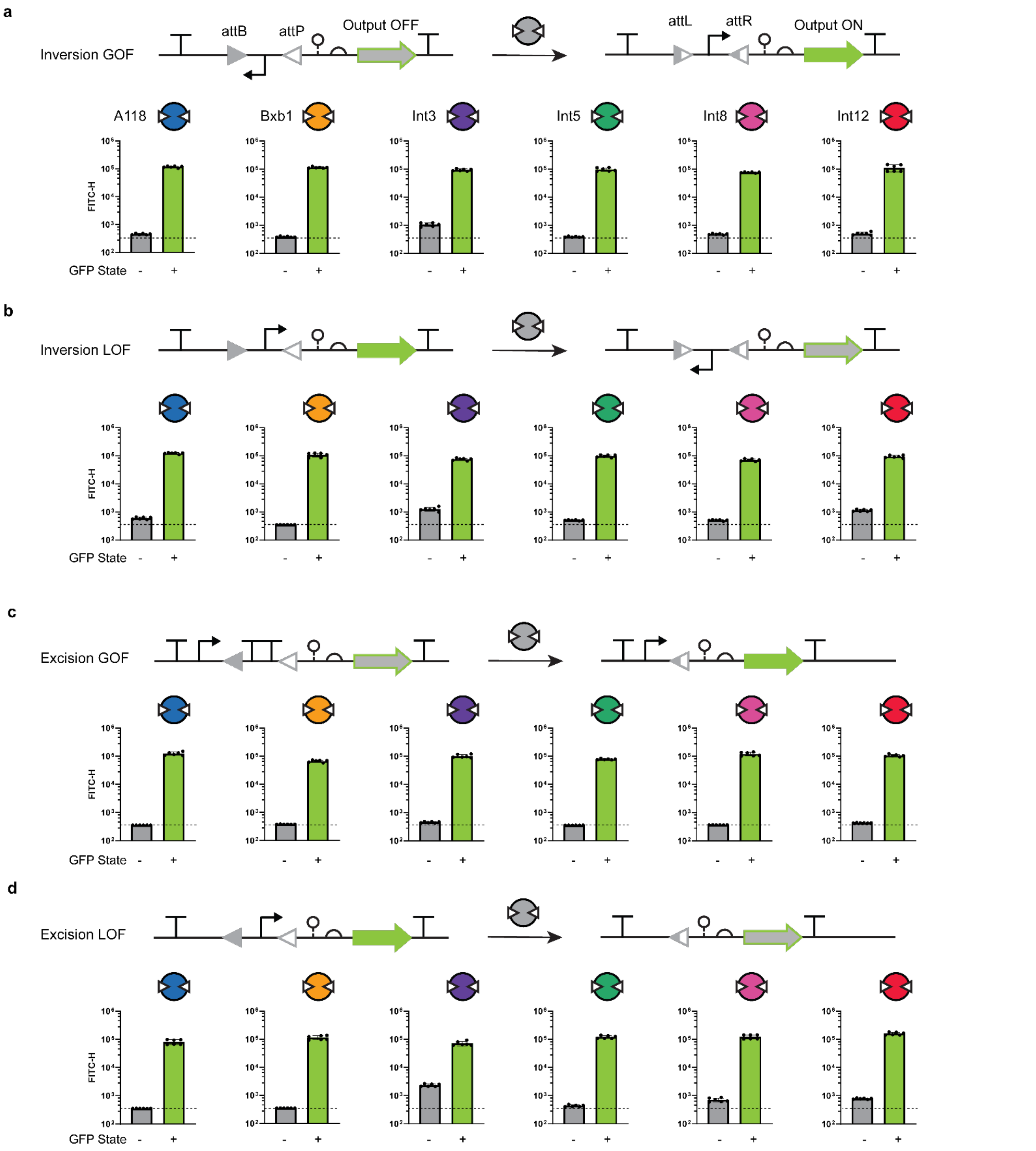

Supplementary Fig. 4 | GFP fluorescence in 24 recombinase circuits. **a** The FITC-H values are shown for the inversion GOF circuits. **b** The FITC-H values are shown for the inversion LOF circuits. **c** The FITC-H values are shown for the excision GOF circuits. **d** The FITC-H values are shown for the excision LOF circuits. The dashed line represents the autofluorescence of wild-type *E. coli* harboring no plasmids. The FITC-H values in **a-d** correspond to the population data presented in Fig. 3b-e. Source data are provided as a Source Data file. Data represent the average of $n = 6$ biological replicates, with groups of three taken on two separate days. Error bars correspond to the SEM of these measurements.

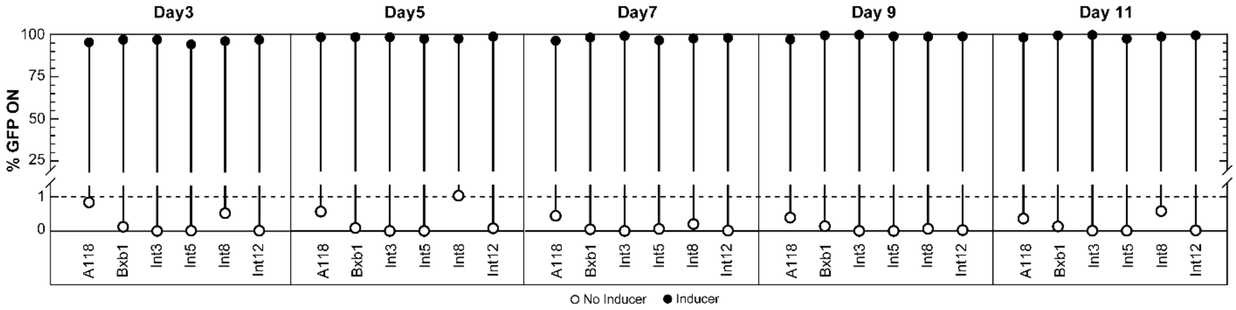

Supplementary Fig. 5 | Additional data related to the genetic stability of EcMem. Data from a second evolutionary trajectory are shown from the experiment presented in Fig. 3f. The EcMem strain transformed with each of the six inversion GOF circuits is cultured continuously for 11 days. Every other day the cultures are used to seed media with inducers to assess for maintenance of recombinase functionality. Open circles represent no inducer, and filled circles represent induced cultures.

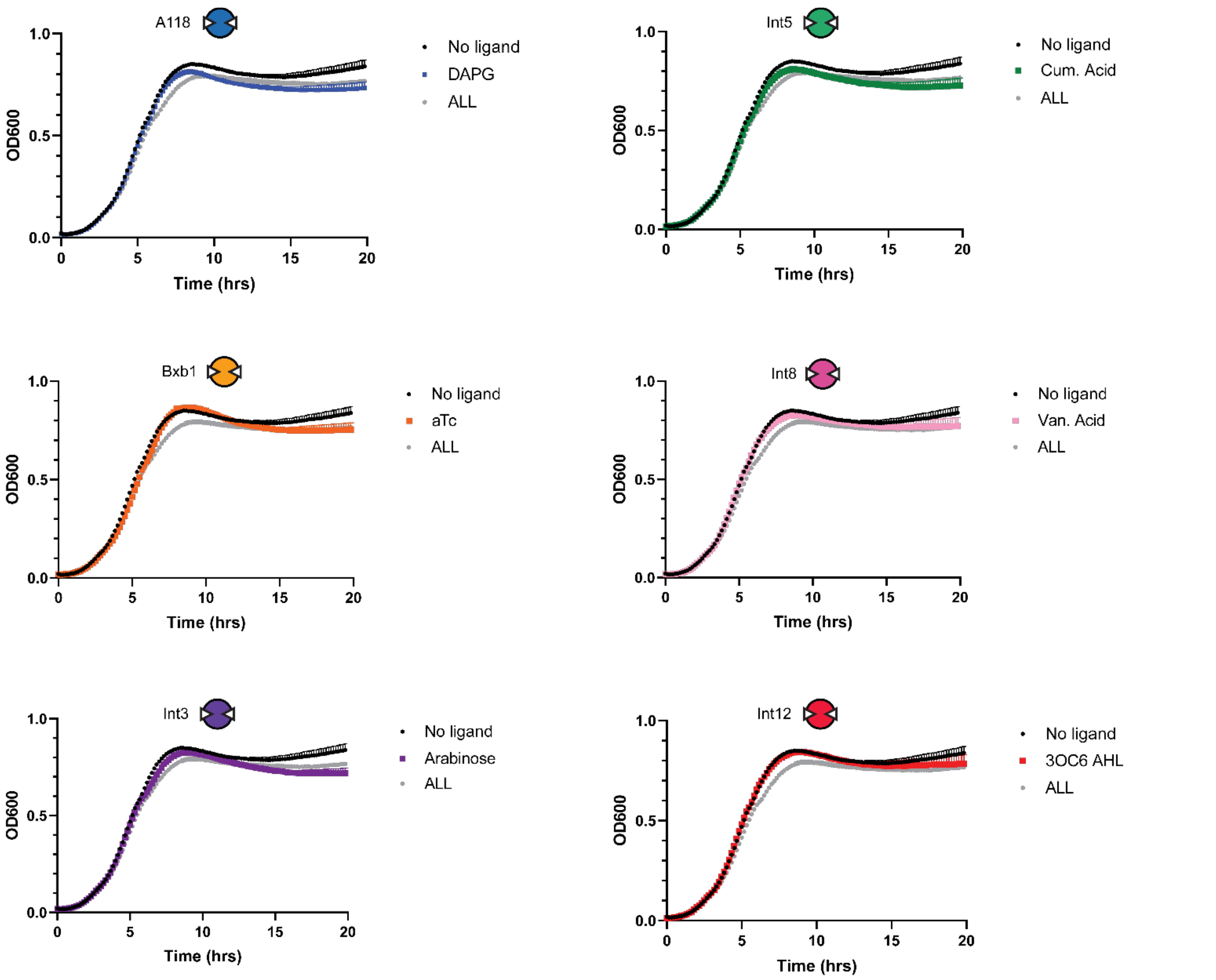

Supplementary Fig. 6 | Cellular burden of recombinase expression. Growth curves of EcMem in minimal media during specific recombinase expression are shown. Each graph shows the growth curve for the EcMem without inducers (black), with all inducers (grey), and with the specific inducer of the indicated recombinase (color). Source data are provided as a Source Data file. Data represent the average of $n = 6$ biological replicates, with groups of three taken on two separate days. Error bars correspond to the SEM of these measurements.

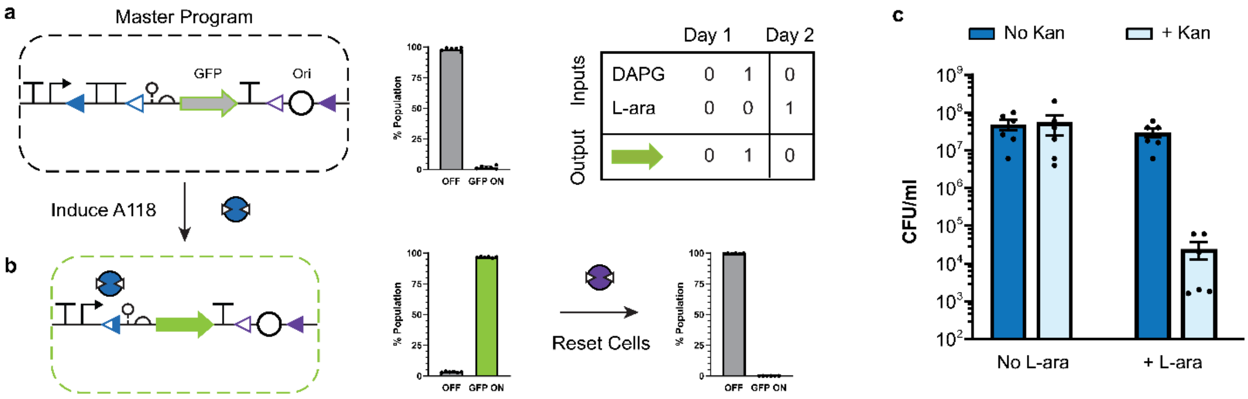

Supplementary Fig. 7 | A118 excision gain-of-function with cellular reset. a The A118 excision GOF circuit is shown with the Int3 origin excision (left). The percentage of recombined cells after growth in MM without inducer is shown (middle) along with an input-output table for this program (right). **b** The recombined circuit is shown after A118 induction (left). The percentage of recombined cells after growth in MM with DAPG is shown (middle). Cells were then grown with L-ara to induce Int3 expression and origin excision (right). **c** Efficiency of the cellular reset is shown. Reset cells from **b** were serially diluted and plated on LB agar with and without kanamycin to assess for pSC101 plasmid loss. Source data are provided as a Source Data file. Data represent the average of *n* = 6 biological replicates, with groups of three taken on two separate days. Error bars correspond to the SEM of these measurements.

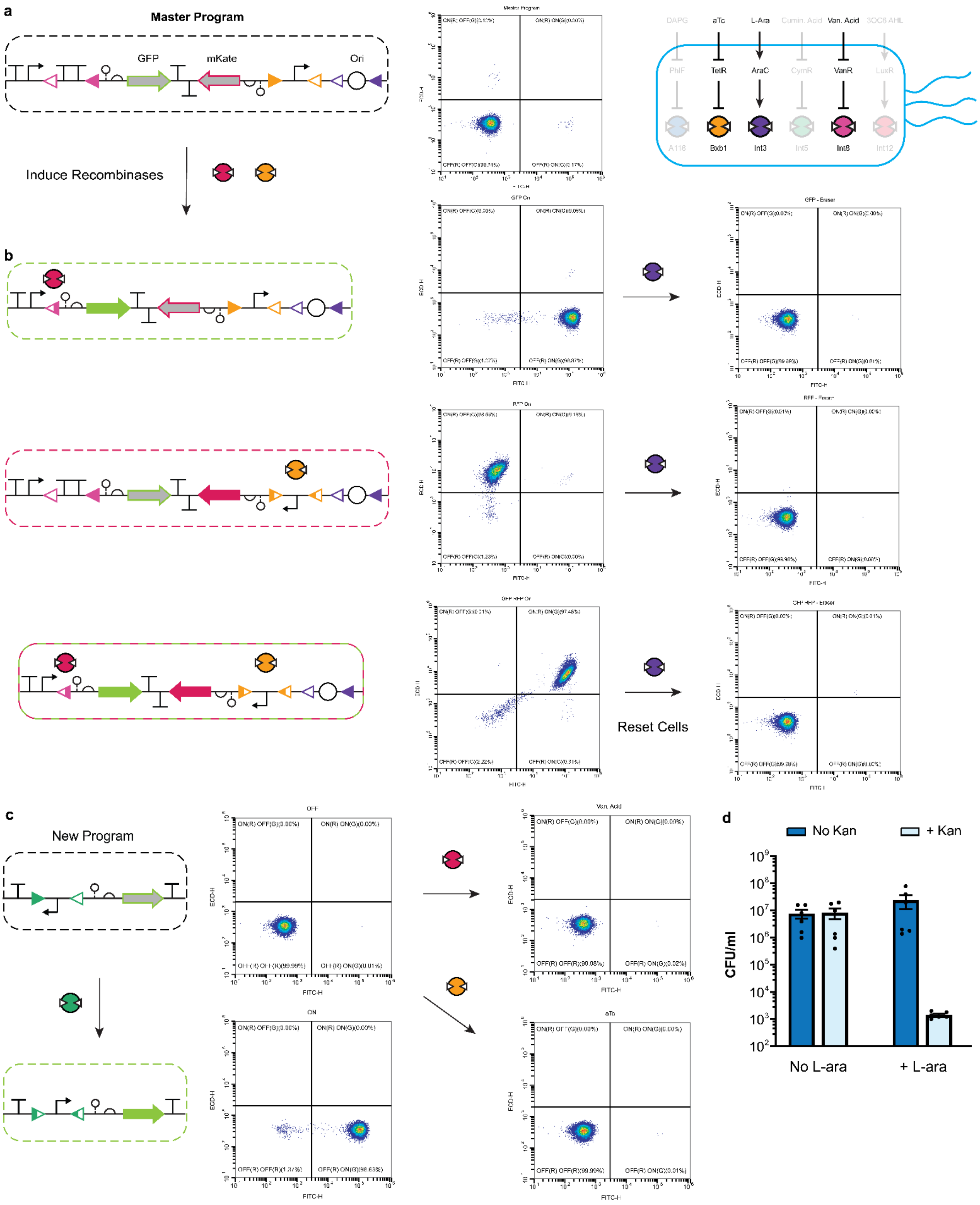
a
Master Program
GFP
mKate
Ori
Induce Recombinases
DAPG
aTc
L-Ara
Cumin. Acid
Van. Acid
3OC6 AHL
PhlF
TetR
AraC
CymR
VanR
LuxR
A118
Bxb1
Int3
Int5
Int8
Int12
b
Reset Cells
c
New Program
d
No Kan
+ Kan
CFU/ml
No L-ara
+ L-ara
FITC-H
ECD-H

Supplementary Fig. 8 | Extended data related to Figure 4. **a** Fig. 4a is recreated with flow cytometry dot plots. **b** Fig. 4b is recreated with flow cytometry dot plots. **c** Fig. 4d is recreated with flow cytometry dot plots. FITC-H is shown on the X-axis representing GFP expression and ECD-H is shown on the Y-axis representing mKate expression. A single representative dot plot is shown for each case. **d** Efficiency of the cellular reset is shown. Reset cells from the "GFP + mKate" state in **b** were serially diluted and plated on LB agar with and without kanamycin before and after Int3 induction to assess for pSC101 plasmid loss. Resultant colonies were counted, and colony-forming units (CFU) were determined. Source data are provided as a Source Data file. Data in **d** represent the average of $n = 6$ biological replicates, with groups of three taken on two separate days. Error bars correspond to the SEM of these measurements.

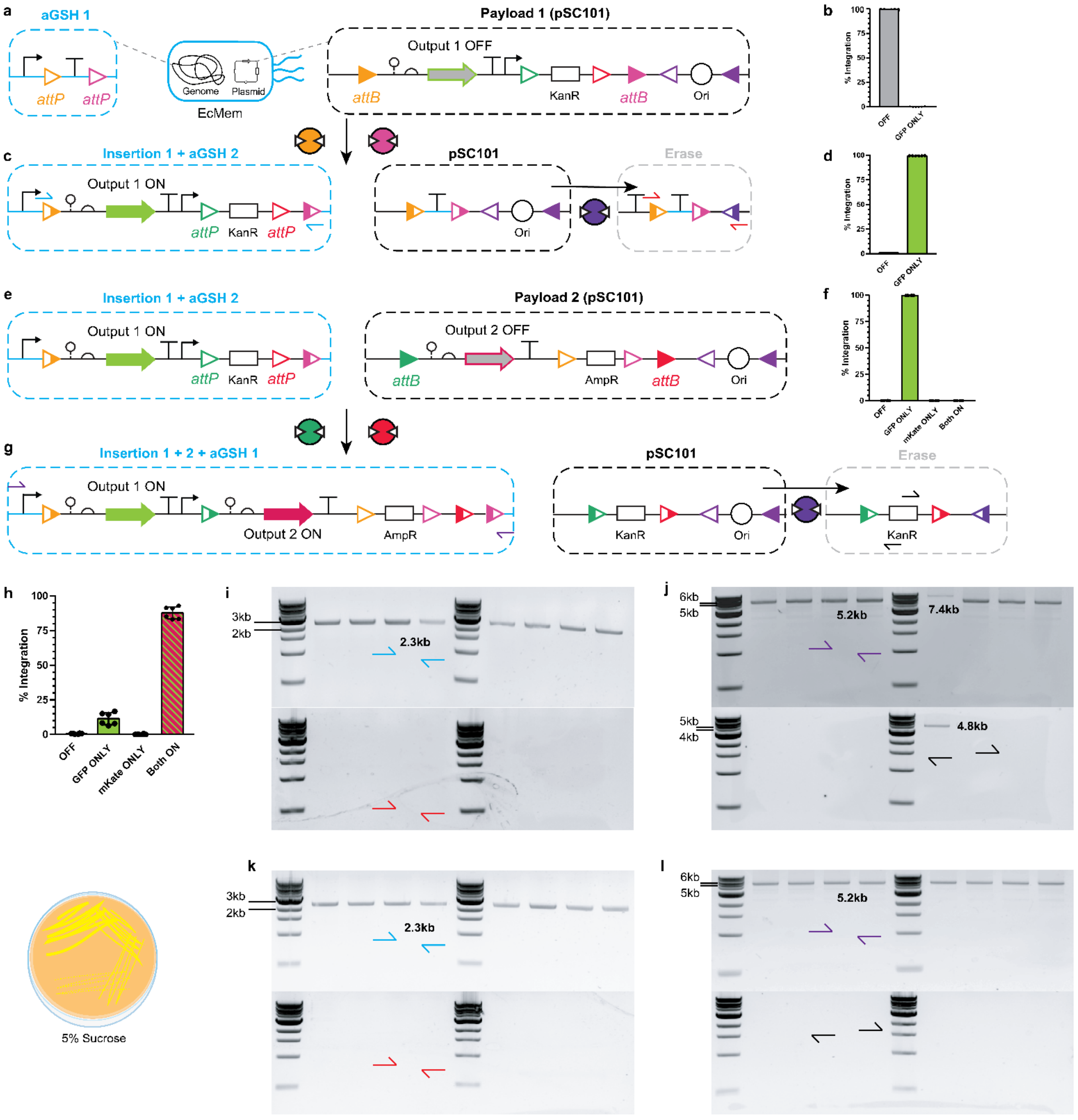

Supplementary Fig. 9 | Data related to inducible integration. **a** The first genomic safe harbor (aGSH1) recording site (left) and the memory sequence (right) are shown (note that SacB is not present in this version). **b** The percentage of cells expressing GFP prior to recombinase induction is shown. **c** The recombined genomic DNA (left) and plasmid DNA (middle) states are shown after induction of Bxb1 and Int8, followed by Int3 induction to erase the plasmids (right). **d** The percentage of cells expressing GFP after recombinase induction is shown, representing the percentage of integration. **e** The new genomic sequence and aGSH2 (left) are shown with the second memory sequence (right). **f** The percentage of cells expressing GFP and mKate prior to recombinase induction is shown. **g** The recombined genomic DNA (left) and plasmid DNA (middle) states are shown after induction of Int5 and Int12, followed by Int3 induction (right). **h** The percentage of cells expressing GFP and mKate after recombinase induction is shown. **i** Representative colony PCR products of 8 colonies from **c** are shown. Primers are denoted

by colored half arrows. **j** Representative colony PCR products of 8 colonies from **g** are shown. **k** Representative colony PCR products of 8 colonies from Fig. 5c are shown. **l** Representative colony PCR products of 8 colonies from Fig. 5g are shown. Source data are provided as a Source Data file. Data in **b, d, f,** and **h** represent the average of $n = 6$ biological replicates, with groups of three taken on two separate days. Error bars correspond to the SEM of these measurements.

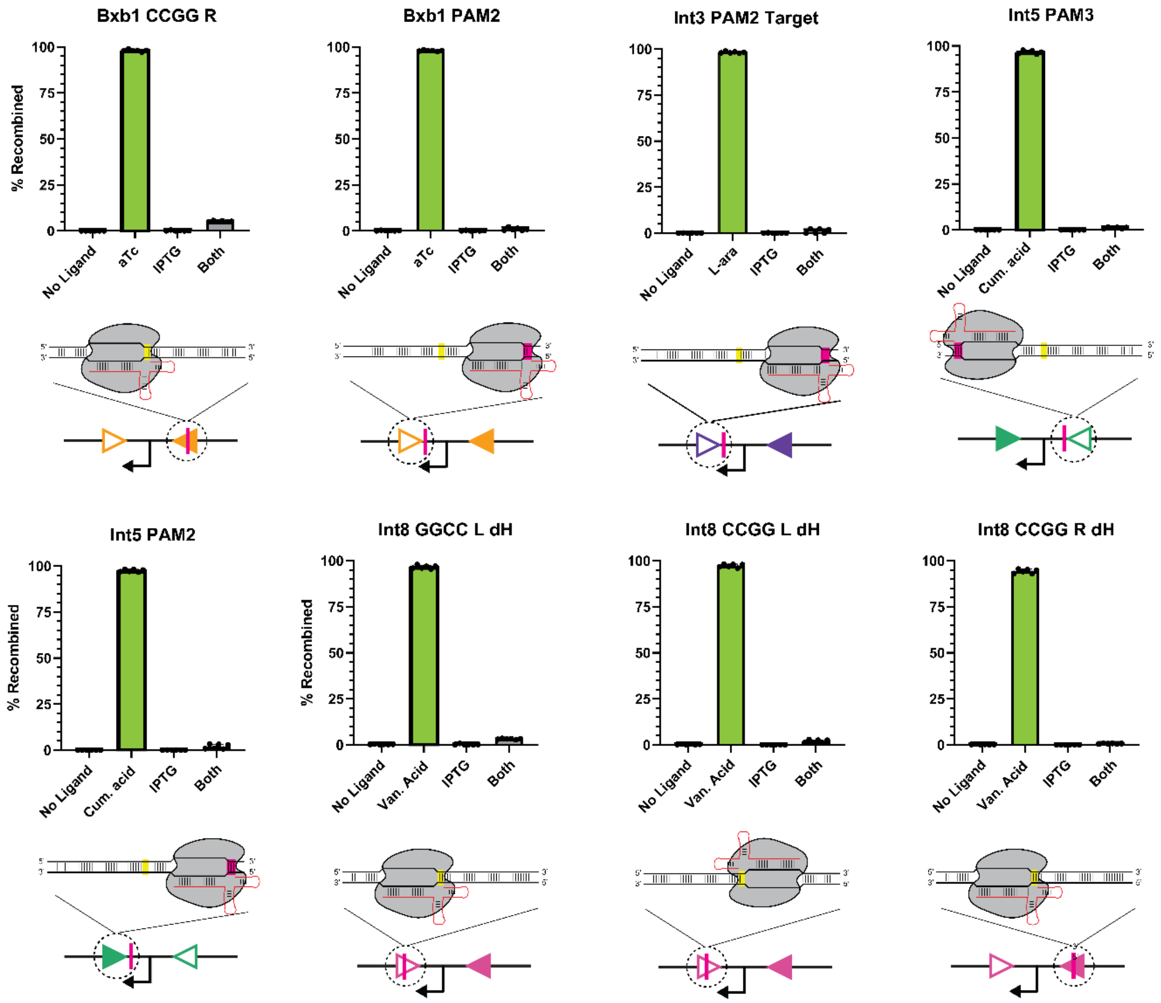

Supplementary Fig. 10 | Additional demonstrations of CRISPR protection. Additional examples of successful CRISPR protection are shown. Cells transformed with the circuit described in Fig. 6a were assayed for recombination performance with cognate inducer as well as in the presence of IPTG. The specific sgRNA target is shown below each bar graph. Source data are provided as a Source Data file. Data represent the average of n = 6 biological replicates, with groups of three taken on two separate days. Error bars correspond to the SEM of these measurements.

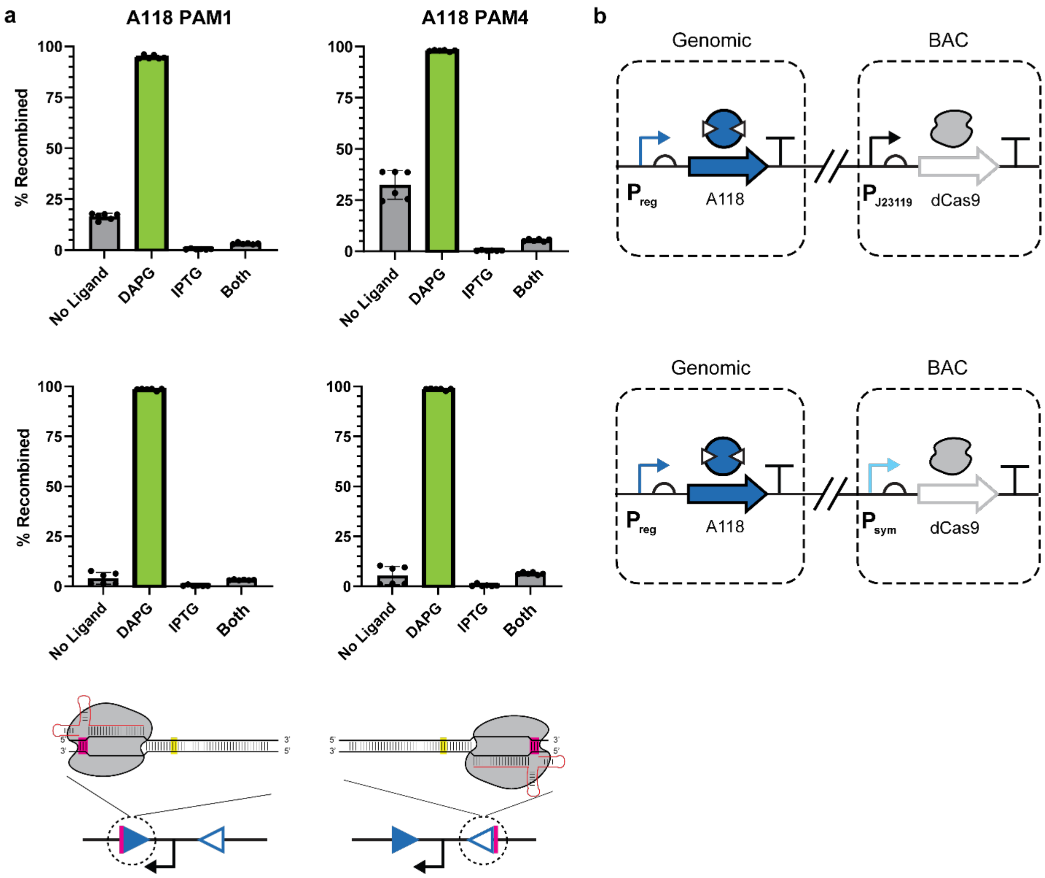

Supplementary Fig. 11 | dCas9 affects PhlF- and A118-based circuits. a The circuit described in Fig. 6a is tested with A118. The top bar graphs show circuit performance when dCas9 is constitutively expressed from the BAC while the bottom bar graphs show circuit performance when dCas9 is controlled by an IPTG-inducible promoter (P_{sym}). **b** The genetic context for recombinase and dCas9 expression is shown next to the appropriate bar graphs. Circuits were assayed in the EcMem strain. Source data are provided as a Source Data file. Data represent the average of n = 6 biological replicates, with groups of three taken on two separate days. Error bars correspond to the SEM of these measurements.

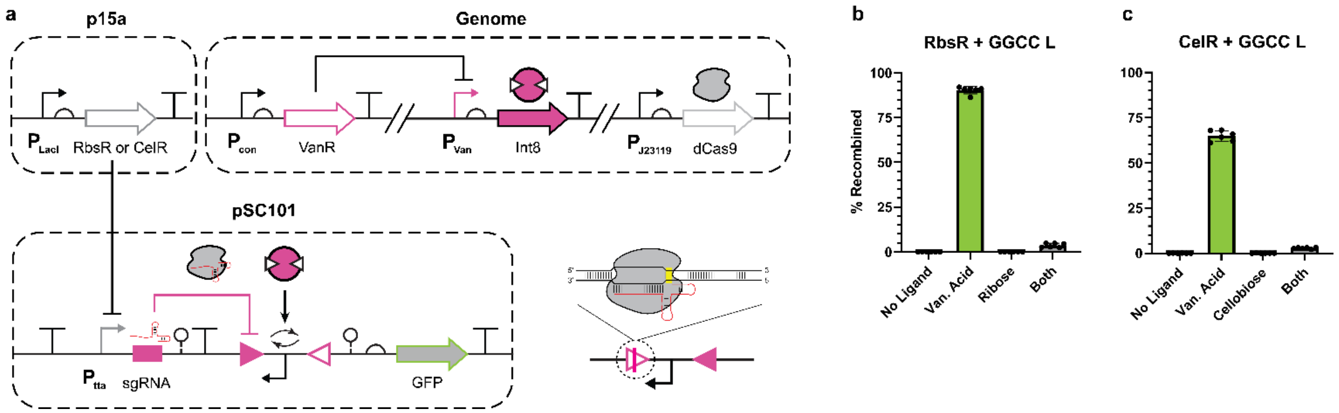

Supplementary Fig. 12 | Controlling CRISPRp with additional transcription factors. a CRISPRp programs using transcription factors harbored on a p15a plasmid are shown. RbsR or CelR regulates sgRNA production from the P_{tta} promoter of varying strengths (bH for RbsR and cG for CelR, see Supplementary Data Files 1 and 3 for details). **b** The performance of RbsR-controlled CRISPRp is shown. **c** The performance of CelR-controlled CRISPRp is shown. Circuits were assayed in the EcMem strain. Source data are provided as a Source Data file. Data represent the average of n = 6 biological replicates, with groups of three taken on two separate days. Error bars correspond to the SEM of these measurements.

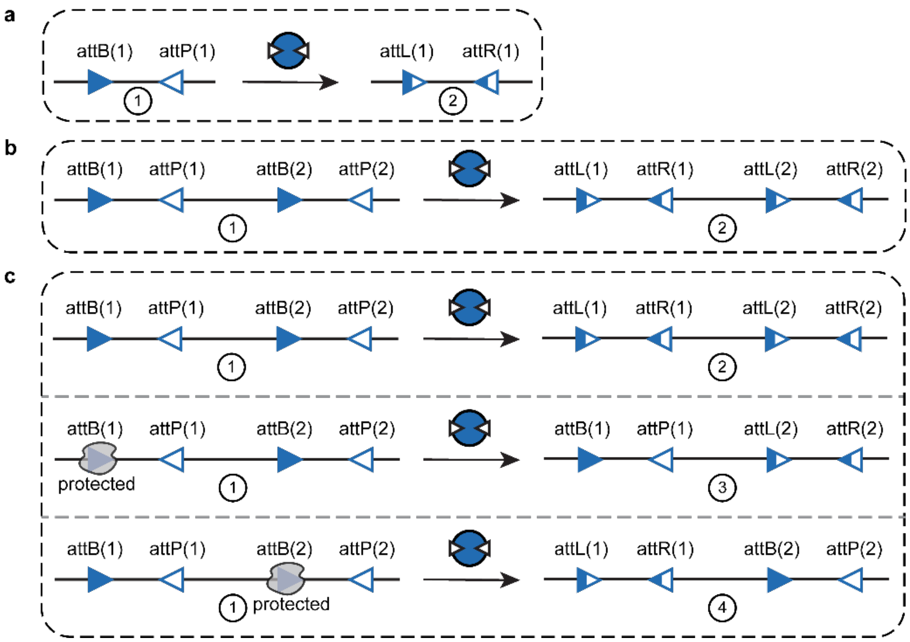

Supplementary Fig. 13 | Expansion of memory capacity using CRISPRp. a Given one set of *att* sites to recombine, a single recombinase allows for $n = 2$ distinct DNA states. **b** Given two sets of *att* sites to recombine (with orthogonal dinucleotide cores), a single recombinase still only allows for $n = 2$ distinct DNA states (assuming complete recombination). **c** Given the same two sets of *att* sites as in **b**, along with the ability to apply CRISPRp to *att* sites independently, a single recombinase now allows for $n = 4$ distinct DNA states. If three sets of *att* sites are used, a single recombinase can produce $n = 9$ distinct DNA states. In theory, for n sets of orthogonal *att* sites, there are n^2 possible distinct DNA states.

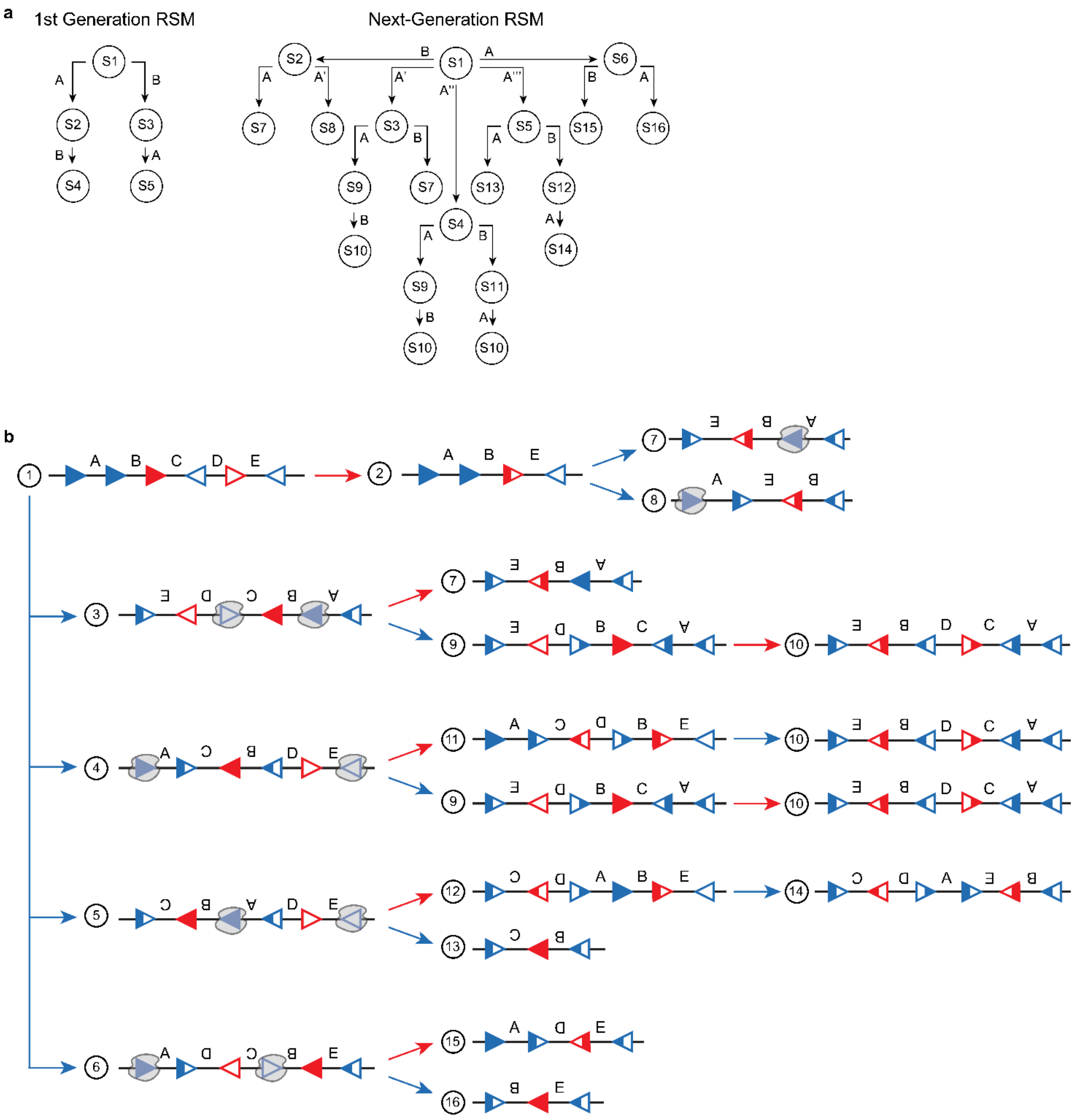

Supplementary Fig. 14 | Next-generation RSM design. a A typical 2-input RSM designed by Roquet *et al.* is shown[8] (left). The expanded RSM state diagram is shown if CRISPRp is applied (right). "A" and "B" represent inputs for different recombinases. A ' indicates a unique recombination event due to the ability to selectively use CRISPRp. **b** The genetic schematic for **a** is shown. Red arrows indicate recombination by recombinase 1 and blue arrows indicate recombination by recombinase 2. A grey lobe covering an *att* site denotes programmed CRISPRp of that site. This RSM assumes that CRISPRp can be applied to any *att* site independently.

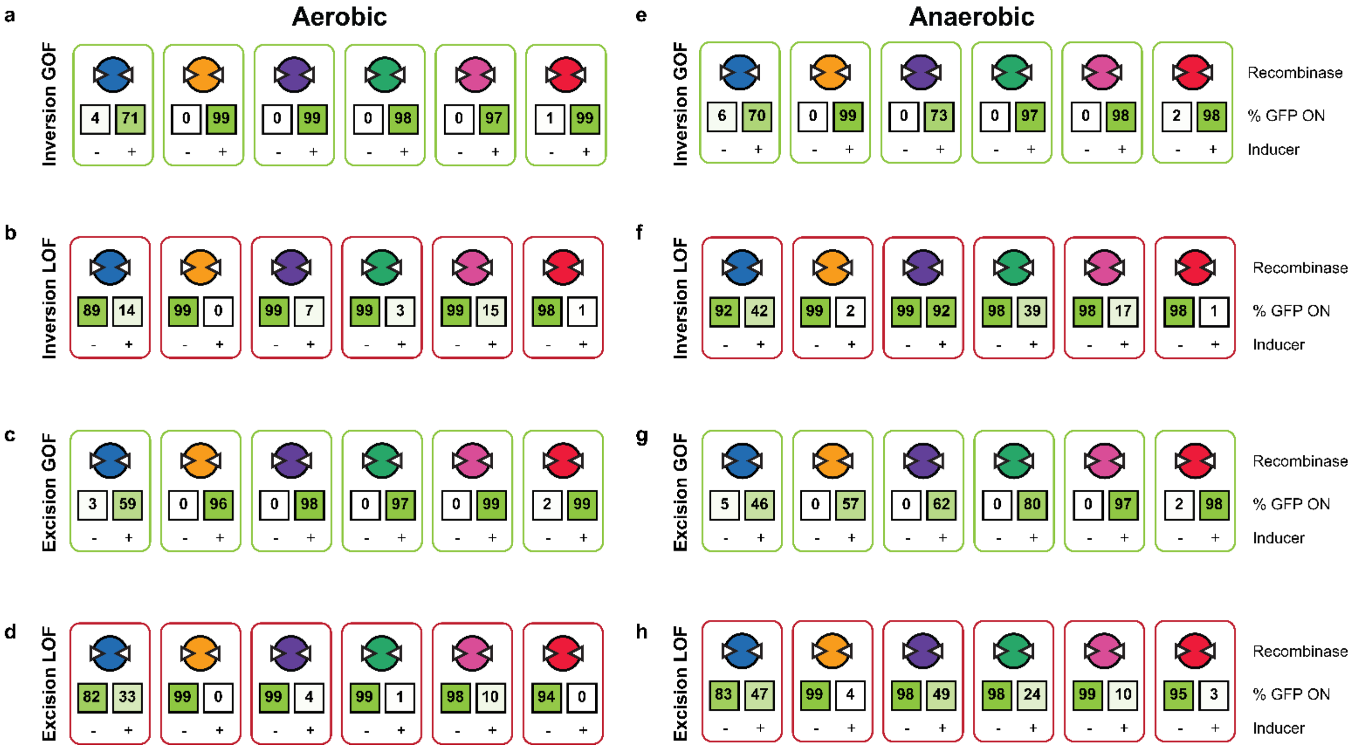

Supplementary Fig. 15 | Performance of recombinase circuits in EcMem^{Pro}. a The performances of the inversion GOF circuits assayed aerobically in EcMem^{Pro} are shown. **b** The performances of the inversion LOF circuits assayed aerobically in EcMem^{Pro} are shown. **c** The performances of the excision GOF circuits assayed aerobically in EcMem^{Pro} are shown. **d** The performances of the excision LOF circuits assayed aerobically in EcMem^{Pro} are shown. **e** The performances of the inversion GOF circuits assayed anaerobically in EcMem^{Pro} are shown. **f** The performances of the inversion LOF circuits assayed anaerobically in EcMem^{Pro} are shown. **g** The performances of the excision GOF circuits assayed anaerobically in EcMem^{Pro} are shown. **h** The performances of the excision LOF circuits assayed anaerobically in EcMem^{Pro} are shown. Source data are provided as a Source Data file. Data represent the average of n = 6 biological replicates, with groups of three taken on two separate days. Aerobic circuits were assayed in M9 minimal medium. Anaerobic circuits were assayed in TYG broth (see Methods).

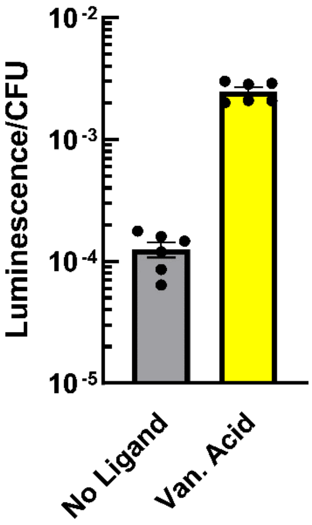

Supplementary Fig. 16 | VanR regulation of Nanoluc in *B. thetaiotaomicron.* The performance of the VanR biosensor is shown. *B. thetaiotaomicron* was grown in TYG broth with and without van. acid and assayed for luminescence (Methods). Source data are provided as a Source Data file. Data represent the average of n = 6 biological replicates, with groups of three taken on two separate days. Error bars correspond to the SEM of these measurements.

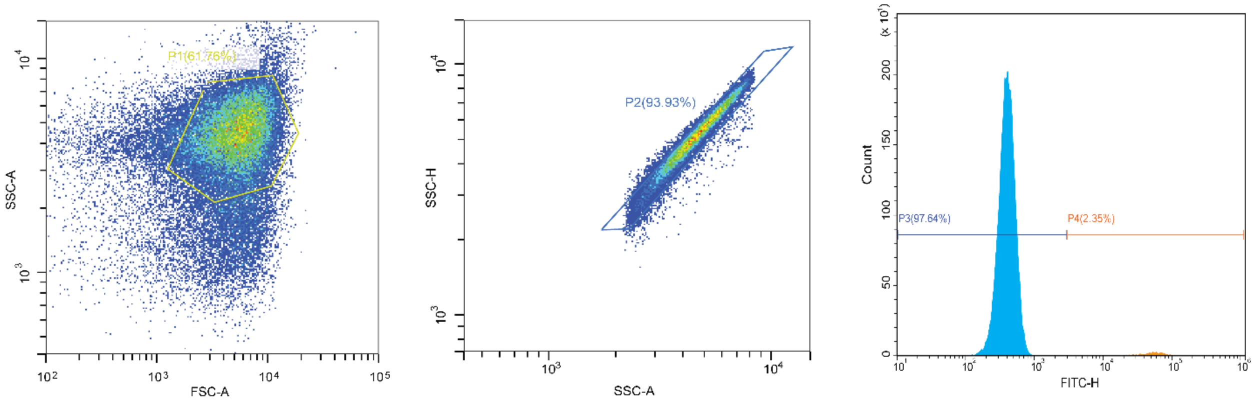

Supplementary Fig. 17 | Example gating strategy for flow cytometry. Representative gates used in flow cytometry analysis are shown. Cells were first gated by side scatter area vs. forward scatter area (left). This population was then gated by side scatter height vs. side scatter area to discriminate single cells (middle). In this example, cells with a FITC-H value greater than 3E3 were deemed GFP-positive while cells with a lower FITC-H value were deemed GFP-negative.

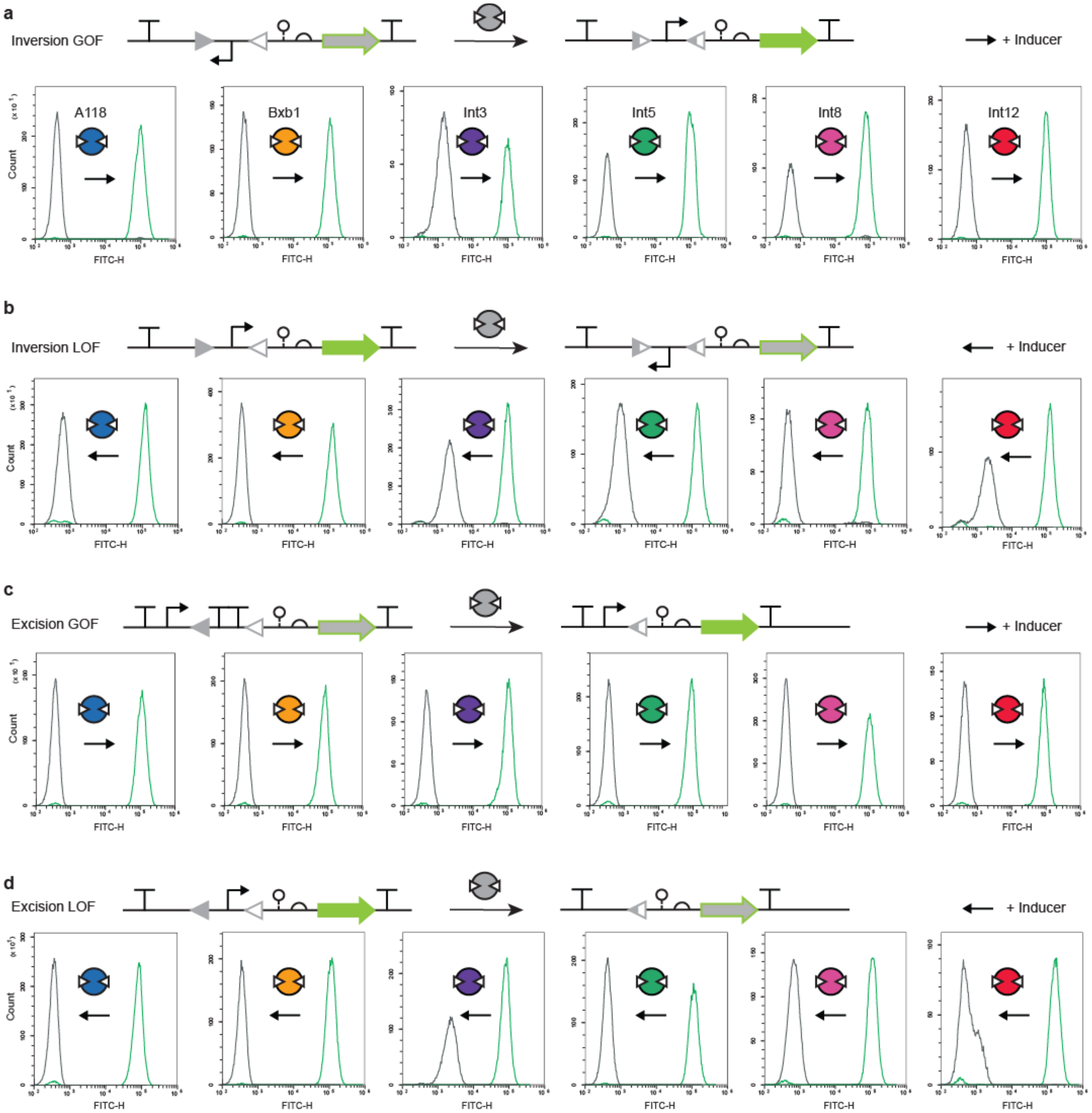

Supplementary Fig. 18 | Representative flow cytometry data for 24 recombinase circuits. A representative flow cytometry plot is provided for each of the inversion GOF circuits **a**, the inversion LOF circuits **b**, the excision GOF circuits **c**, and the excision LOF circuits **d**. The uninduced and induced states are shown on the same plot with an arrow denoting the transition of the populations upon induction. The y-axis is the cell count and the x-axis is the GFP intensity (FITC-H).

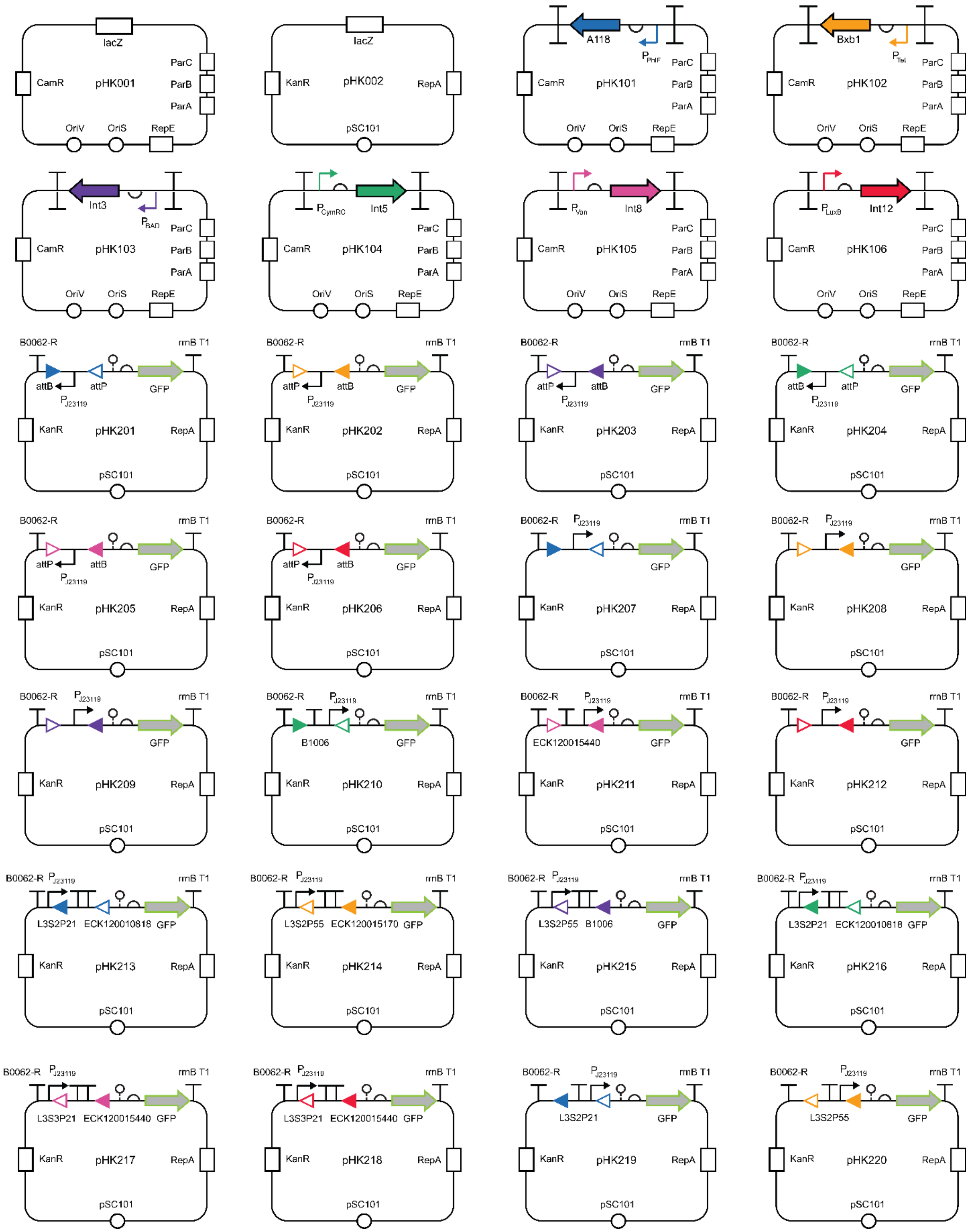

Supplementary Fig. 19 | Relevant plasmid maps used in this study part 1. Maps correspond to descriptions in Supplementary Data File 3.

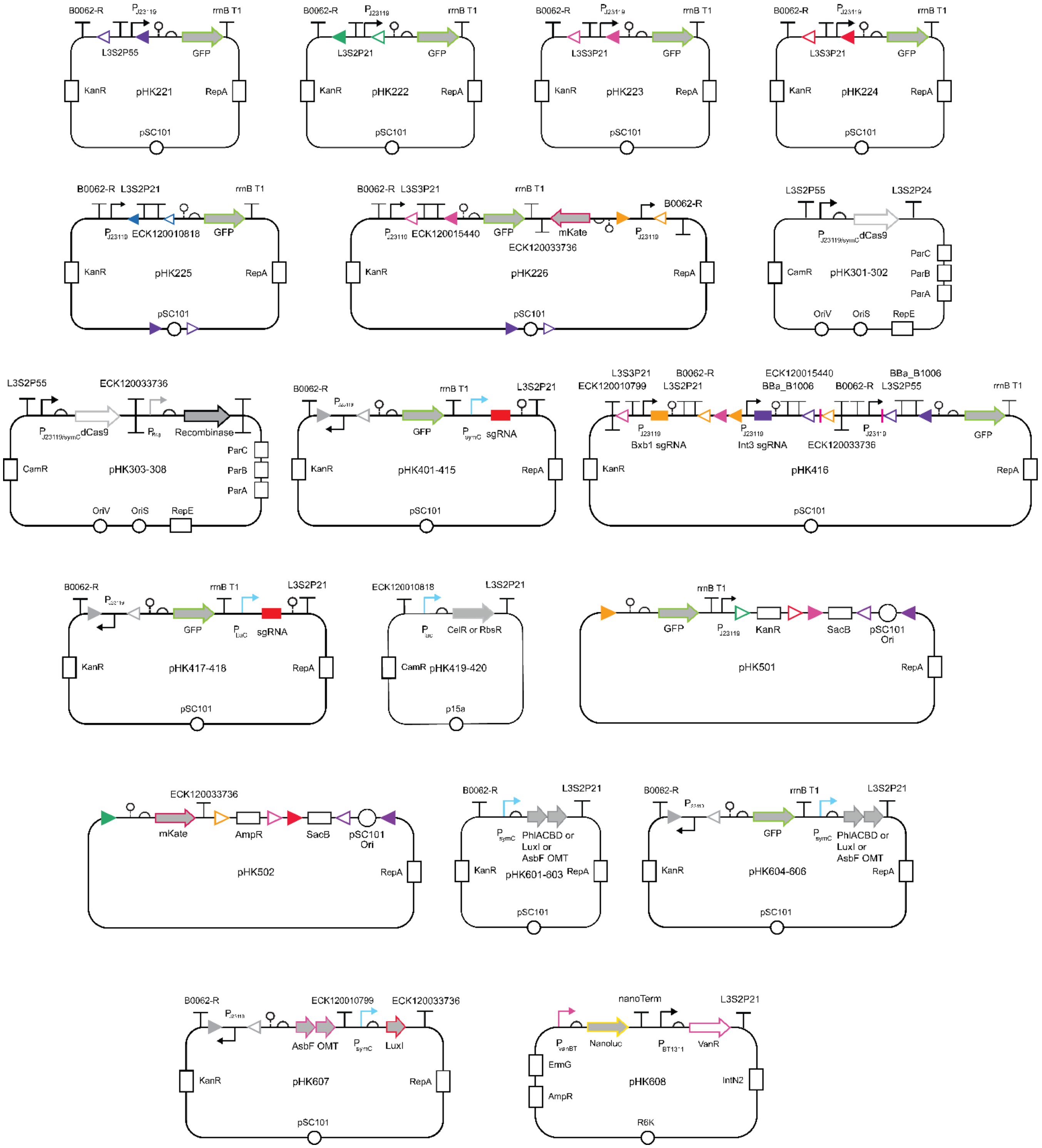

Supplementary Fig. 20 | Relevant plasmid maps used in this study part 2. Maps correspond to descriptions in Supplementary Data File 3.

Supplementary Table 1: Primers used for genomic integration confirmation

**Primer Name**	**Sequence 5' to 3'**
CP1INT_fwd	GTCGGGGTTTGTACCGTACACCAC
CP1INT_rev	TTAATAAACTATGGAAGTATGTACAGTCTTGC
CP1ERA_fwd	GCCCGGATGATCCTGACGAC
CP1ERA_rev	GTTTGTAAAGGAGACTGATAATGGC
CP2INT_fwd	ATCCGCAGGCAAGCGAAGATG
CP2INT_rev	GTTGAGGATTTTCGCATTCGG
CP2ERA_fwd	TGGATACTTTCTCGGCAGGAG
CP2ERA_rev	TCATGGCTGATGCAATGCG

Supplementary References

1. Short, A.E., Kim, D., Milner, P.T. & Wilson, C.J. Next generation synthetic memory *via* intercepting recombinase function. *Nat Commun* **14**, 5255 (2023).
2. Nielsen, A.A. et al. Genetic circuit design automation. *Science* **352**, aac7341 (2016).
3. Jinek, M. et al. A programmable dual-RNA-guided DNA endonuclease in adaptive bacterial immunity. *Science* **337**, 816-821 (2012).
4. Sternberg, S.H., Redding, S., Jinek, M., Greene, E.C. & Doudna, J.A. DNA interrogation by the CRISPR RNA-guided endonuclease Cas9. *Nature* **507**, 62-67 (2014).
5. Thyme, S.B., Akhmetova, L., Montague, T.G., Valen, E. & Schier, A.F. Internal guide RNA interactions interfere with Cas9-mediated cleavage. *Nat Commun* **7**, 11750 (2016).
6. Li, Y., Teng, X., Zhang, K., Deng, R. & Li, J. RNA Strand Displacement Responsive CRISPR/Cas9 System for mRNA Sensing. *Anal Chem* **91**, 3989-3996 (2019).
7. Riesenberg, S., Helmbrecht, N., Kanis, P., Maricic, T. & Pääbo, S. Improved gRNA secondary structures allow editing of target sites resistant to CRISPR-Cas9 cleavage. *Nat Commun* **13**, 489 (2022).
8. Roquet, N., Soleimany, A.P., Ferris, A.C., Aaronson, S. & Lu, T.K. Synthetic recombinase-based state machines in living cells. *Science* **353**, aad8559 (2016).
