# Supplementary material for: Engineering intelligent chassis cells via recombinase-based MEMORY circuits
Source: Nat Commun. 2024 Mar 18;15:2418. doi: 10.1038/s41467-024-46755-1 (PMC10948884; doi:10.1038/s41467-024-46755-1)
Supplement: Supplementary file 3 — Description of Additional Supplementary Files [file 41467_2024_46755_MOESM3_ESM.pdf]

Description of Supplementary Files

Supplementary Data 1: Sequences of genetic parts used in this work

Supplementary Data 2: Genomic sequence of the EcMem recombinase array

Supplementary Data 3: Plasmids created in this study
