# Supplementary material for: Engineering intelligent chassis cells via recombinase-based MEMORY circuits
Source: Nat Commun. 2024 Mar 18;15:2418. doi: 10.1038/s41467-024-46755-1 (PMC10948884; doi:10.1038/s41467-024-46755-1)
Supplement: Supplementary file 7 — Reporting Summary [file 41467_2024_46755_MOESM7_ESM.pdf]

nature portfolio

Corresponding author(s): Corey J. Wilson

Last updated by author(s): Feb 27, 2024

Reporting Summary

Nature Portfolio wishes to improve the reproducibility of the work that we publish. This form provides structure for consistency and transparency in reporting. For further information on Nature Portfolio policies, see our Editorial Policies and the Editorial Policy Checklist.

Statistics

For all statistical analyses, confirm that the following items are present in the figure legend, table legend, main text, or Methods section.

n/a	Confirmed	
☐	☒	The exact sample size (*n*) for each experimental group/condition, given as a discrete number and unit of measurement
☐	☒	A statement on whether measurements were taken from distinct samples or whether the same sample was measured repeatedly
☐	☒	The statistical test(s) used AND whether they are one- or two-sided<br>*Only common tests should be described solely by name; describe more complex techniques in the Methods section.*
☒	☐	A description of all covariates tested
☐	☒	A description of any assumptions or corrections, such as tests of normality and adjustment for multiple comparisons
☐	☒	A full description of the statistical parameters including central tendency (e.g. means) or other basic estimates (e.g. regression coefficient) AND variation (e.g. standard deviation) or associated estimates of uncertainty (e.g. confidence intervals)
☐	☒	For null hypothesis testing, the test statistic (e.g. *F*, *t*, *r*) with confidence intervals, effect sizes, degrees of freedom and *P* value noted<br>*Give P values as exact values whenever suitable.*
☒	☐	For Bayesian analysis, information on the choice of priors and Markov chain Monte Carlo settings
☒	☐	For hierarchical and complex designs, identification of the appropriate level for tests and full reporting of outcomes
☒	☐	Estimates of effect sizes (e.g. Cohen's *d*, Pearson's *r*), indicating how they were calculated

Our web collection on statistics for biologists contains articles on many of the points above.

Software and code

Policy information about availability of computer code

Data collection	Molecular Devices Spectramax M2e plate reader, Beckman Coulter CytoFLEX S Flow Cytometer, ChemiDoc XRS+ (Bio-Rad)
Data analysis	Microsoft Excel, ApE Plasmid Editor v3.0.6, Graphpad Prism 9.3.1, CytExpert 2.4, Molecular Devices SoftMax Pro (version 7.0.3), Image Lab 6.0.1

For manuscripts utilizing custom algorithms or software that are central to the research but not yet described in published literature, software must be made available to editors and reviewers. We strongly encourage code deposition in a community repository (e.g. GitHub). See the Nature Portfolio guidelines for submitting code & software for further information.

Data

Policy information about availability of data

All manuscripts must include a data availability statement. This statement should provide the following information, where applicable:

- Accession codes, unique identifiers, or web links for publicly available datasets
- A description of any restrictions on data availability
- For clinical datasets or third party data, please ensure that the statement adheres to our policy

The authors declare that all data supporting the findings of this study are available within the paper and its supplementary information. The analyzed data and source data will be made available in Supplementary Data Files and Source Data. Any other information can be made available from the corresponding author upon reasonable request. The sequences of the plasmids used in this study are available from Genbank with accession numbers PP125204-PP125275.

Research involving human participants, their data, or biological material

Policy information about studies with human participants or human data. See also policy information about sex, gender (identity/presentation), and sexual orientation and race, ethnicity and racism.

Reporting on sex and gender	NA
Reporting on race, ethnicity, or other socially relevant groupings	NA
Population characteristics	NA
Recruitment	NA
Ethics oversight	NA

Note that full information on the approval of the study protocol must also be provided in the manuscript.

Field-specific reporting

Please select the one below that is the best fit for your research. If you are not sure, read the appropriate sections before making your selection.

☒ Life sciences ☐ Behavioural & social sciences ☐ Ecological, evolutionary & environmental sciences

For a reference copy of the document with all sections, see nature.com/documents/nr-reporting-summary-flat.pdf

Life sciences study design

All studies must disclose on these points even when the disclosure is negative.

Sample size	A sample size of n=6 was chosen for all assays except for the experiments described in Fig. 1f and 3c. The sample size was based on previous works (Rondon et al., 2019), (Groseclose et al., 2020) and comparison with standard techniques in literature (typical n=3 biological replicates). For the evolutionary stability experiment (Fig. 1f), n=2 independent trials were performed concurrently. For the cellular reset and re-transformation experiment (Fig. 3c), n=3 biological replicates were assayed on a single day.
Data exclusions	No data has been excluded from analyses. All data collected is shown on plots and is included in the Source Data file.
Replication	All attempts at replication were successful. All experiments with n=6 replicates were performed on two separate days with n=3 biological replicates per day.
Randomization	Randomization was not performed in this study as it was not relevant. Knowledge of genotype was required during experiments to ensure that data generated could be analyzed appropriately. Cultures were always started from single colonies derived from known strains or transformants.
Blinding	Blinding was not relevant to this study since knowledge of genotype and plasmid sequence was required for experiments to be performed under the correct conditions.

Reporting for specific materials, systems and methods

We require information from authors about some types of materials, experimental systems and methods used in many studies. Here, indicate whether each material, system or method listed is relevant to your study. If you are not sure if a list item applies to your research, read the appropriate section before selecting a response.

Materials & experimental systems

n/a	Involved in the study	
☒	☐	Antibodies
☒	☐	Eukaryotic cell lines
☒	☐	Palaeontology and archaeology
☒	☐	Animals and other organisms
☒	☐	Clinical data
☒	☐	Dual use research of concern
☒	☐	Plants

Methods

n/a	Involved in the study	
☒	☐	ChIP-seq
☐	☒	Flow cytometry
☒	☐	MRI-based neuroimaging

Plants

Seed stocks	NA
Novel plant genotypes	NA
Authentication	NA

Flow Cytometry

Plots

Confirm that:

☒ The axis labels state the marker and fluorochrome used (e.g. CD4-FITC).

☒ The axis scales are clearly visible. Include numbers along axes only for bottom left plot of group (a 'group' is an analysis of identical markers).

☒ All plots are contour plots with outliers or pseudocolor plots.

☒ A numerical value for number of cells or percentage (with statistics) is provided.

Methodology

Sample preparation	Samples were prepared as in the Methods section. Briefly, cultures were diluted 1:50 in PBS containing 2 mg/ml kanamycin. Cells were then incubated at room temperature for greater than 1 hour. Cells were then processed by flow cytometry.
Instrument	Beckman Coulter CytoFLEX S
Software	CytExpert Software
Cell population abundance	Cells were not sorted. Greater than 10,000 cells were collected for analysis in all experiments.
Gating strategy	Cell gating is detailed in the Methods section. Briefly, cells were gated by FSC-A vs SSC-A, and then by SSC-H vs SSC-A. For GFP expression experiments, FITC-H was used to deem cells as GFP positive. A typical threshold for GFP expression was a FITC-H value of greater than 3x10^3. For mKate expression experiments, ECD-H was used to deem cells as mKate positive. A typical threshold for mKate expression was an ECD-H value greather than 2x10^3.

☒ Tick this box to confirm that a figure exemplifying the gating strategy is provided in the Supplementary Information.
